# Prediction of postoperative visual acuity in patients with age-related cataracts using macular optical coherence tomography-based deep learning method

**DOI:** 10.3389/fmed.2023.1165135

**Published:** 2023-05-12

**Authors:** Jingwen Wang, Jinhong Wang, Dan Chen, Xingdi Wu, Zhe Xu, Xuewen Yu, Siting Sheng, Xueqi Lin, Xiang Chen, Jian Wu, Haochao Ying, Wen Xu

**Affiliations:** ^1^Eye Center of the Second Affiliated Hospital, School of Medicine, Zhejiang University, Hangzhou, Zhejiang, China; ^2^College of Computer Science and Technology, Zhejiang University, Hangzhou, Zhejiang, China; ^3^Department of Ophthalmology, The First People’s Hospital of Xiaoshan District, Xiaoshan Affiliated Hospital of Wenzhou Medical University, Hangzhou, Zhejiang, China; ^4^Second Affiliated Hospital School of Medicine, School of Public Health, and Institute of Wenzhou, Zhejiang University, Hangzhou, Zhejiang, China; ^5^School of Public Health, Zhejiang University, Hangzhou, Zhejiang, China

**Keywords:** cataract surgery, visual acuity, deep learning, macula, optical coherence tomography

## Abstract

**Background:**

To predict postoperative visual acuity (VA) in patients with age-related cataracts using macular optical coherence tomography-based deep learning method.

**Methods:**

A total of 2,051 eyes from 2,051 patients with age-related cataracts were included. Preoperative optical coherence tomography (OCT) images and best-corrected visual acuity (BCVA) were collected. Five novel models (I, II, III, IV, and V) were proposed to predict postoperative BCVA. The dataset was randomly divided into a training (*n* = 1,231), validation (*n* = 410), and test set (*n* = 410). The performance of the models in predicting exact postoperative BCVA was evaluated using mean absolute error (MAE) and root mean square error (RMSE). The performance of the models in predicting whether postoperative BCVA was improved by at least two lines in the visual chart (0.2LogMAR) was evaluated using precision, sensitivity, accuracy, F1 and area under curve (AUC).

**Results:**

Model V containing preoperative OCT images with horizontal and vertical B-scans, macular morphological feature indices, and preoperative BCVA had a better performance in predicting postoperative VA, with the lowest MAE (0.1250 and 0.1194LogMAR) and RMSE (0.2284 and 0.2362LogMAR), and the highest precision (90.7% and 91.7%), sensitivity (93.4% and 93.8%), accuracy (88% and 89%), F1 (92% and 92.7%) and AUCs (0.856 and 0.854) in the validation and test datasets, respectively.

**Conclusion:**

The model had a good performance in predicting postoperative VA, when the input information contained preoperative OCT scans, macular morphological feature indices, and preoperative BCVA. The preoperative BCVA and macular OCT indices were of great significance in predicting postoperative VA in patients with age-related cataracts.

## 1. Introduction

Cataract, defined as the opacity of the lens, is one of the leading causes of visual impairment worldwide and a primary cause of blindness, estimated to be responsible for 15.2 million cases of blindness in 2020 (1). Many factors lead to the formation of cataracts, including age, diabetes, and ultraviolet irradiation, and age remains the major risk factor for cataracts (2). The only effective treatment is surgery. Most patients can gain excellent visual acuity (VA) after cataract surgeries. However, some patients may fail to obtain satisfying visual outcomes due to complicated fundus diseases. Predicting visual outcomes before cataract surgeries can help patients adjust their expectations appropriately and aid doctors in making reasonable decisions for patients whose vision may not be improved. This can avoid the waste of medical resources and contradictions between doctors and patients.

As the sharpest part of the retina for vision, the macula remains one of the most important factors in determining VA after cataract surgery. Optical coherence tomography (OCT) is a non-invasive, high-resolution cross-sectional imaging modality of the structural retina *in vivo*. The introduction of OCT helps ophthalmologists qualitatively and quantitatively assess the subtle structural changes in the macular region (3, 4). Previous studies have reported that abnormal morphological changes in OCT images can lead to worse visual outcomes in patients with retinal diseases (5–8). Most clinicians currently analyze OCT images empirically to judge the function of the retinal macula and thus roughly estimate the postoperative VA of patients with cataracts. However, there is no standardized evaluation system based on large samples to quantify the relationship between macular morphological changes and postoperative VA of patients with cataracts.

Artificial intelligence (AI), especially deep learning (DL), has been widely used to analyze retinal images in the past few decades. Some studies have achieved satisfactory results in applying DL algorithms to predict postoperative visual outcomes in retinal diseases (9–11). Mao et al. have investigated the predictive factors of VA in patients with retinitis pigmentosa after cataract surgery (12). The preoperative best-corrected visual acuity (BCVA), the status of the external limiting membrane, and central macular thickness are found to be important parameters to predict postoperative VA. Recently, Wei et al. have constructed an OCT-based DL approach to predict the postoperative VA of patients with high myopia (13). Xiang et al. have developed an intelligent system based on OCT images for long-term BCVA prediction in 3 and 5 years after surgery in patients with congenital cataracts (14). All of the above studies have yielded good results. However, these studies are hard to explain the underlying mechanism due to the lack of anatomic and morphological features integrated with the study. It is essential since the microstructure of the macula are closely correlated with the postoperative VA of patients with cataracts (15).

In the present study, AI models were developed based on preoperative macular OCT images and BCVA to predict the postoperative VA in patients with age-related cataracts. The AI models were then compared to evaluate the prediction performances of certain postoperative BCVA and whether postoperative BCVA was improved by at least two lines in the visual chart (0.2LogMAR).

## 2. Materials and methods

### 2.1. Participants

The study was performed on 2,051 eyes from 2,051 patients with cataracts who underwent uneventful cataract surgeries operated by the same experienced cataract surgeon in the Eye Centre at the Second Affiliated Hospital of Zhejiang University, School of Medicine, from December 2018 to June 2020. The dataset consisting of 2051 eyes from 2051 patients with cataracts was randomly divided into a training (*n* = 1,231), validation (*n* = 410), and test set (*n* = 410). Collected clinical data included gender, laterality, and surgical age, as well as BCVA measured preoperatively and 1 month postoperatively. Image data included horizontal and vertical B-scan macular OCT images of the patient at the same preoperative visit.

Inclusion criteria were as follows: (1) age 50–90 years old, diagnosed with senile cataract, the degree of lens opacity was graded by the Lens Opacities Classification System III: cortical opacity at grade 4 and below, posterior subcapsular opacity at grade 4 and below. The hardness of the nucleus was graded by the Emery and Little classification: grade IV and below, (2) reliable OCT measurements of the macula were performed before cataract surgery, (3) underwent peaceful cataract surgeries, and (4) had reliable BCVA measured preoperatively and 1 month postoperatively.

Exclusion criteria were as follows: (1) Amblyopia; (2) Congenital ocular anomalies; (3) Cataracts caused by trauma or congenital anomalies; (4) Refractive media opacities that seriously affected macular OCT image clarity or visual prognosis, such as severe lens opacities, centered corneal opacities, severe vitreous opacities; (5) Poor quality macular OCT images that affected image analysis; (6) Combined with retinal detachment, retinitis pigmentosa, and fundus lesions such as optic nerve and choroid that might affect VA; (7) Combined with nystagmus or head tremor and other diseases that were susceptible to interference during macular OCT examination; (8) Combined with consciousness or intellectual impairment that affected the accuracy of visual acuity test results; The patients are excluded if they meet the any of the above exclusion criteria.

This study was approved by the Institutional Review Board of the Second Affiliated Hospital of Zhejiang University, School of Medicine. Written informed consents was obtained from all the participants. This study complied with the Declaration of Helsinki and was registered at^1^ (accession number NCT04887909).

### 2.2. Macular OCT images

OCT images were acquired from Spectrialis OCT (Heidelberg Engineering, Heidelberg, Germany), Cirrus OCT (Carl Zeiss Meditec, Dublin, California, United States), and FD-OCT (RTVue; Optovue Inc., Fremont, California, USA). Images from Spectrialis OCT had a resolution of 768 by 496 pixels, with a scan width of 10,000 μm and a scan depth of 2,000 μm in the air. Images from Cirrus OCT had a resolution of 938 by 625 pixels, with a scan width of 6,000 μm and a scan depth of 2,000 μm in the air. Images from FD-OCT had a resolution of 1,020 by 960 pixels, with a scan width of 10,000 μm and a scan depth of 2,000 μm in the air.


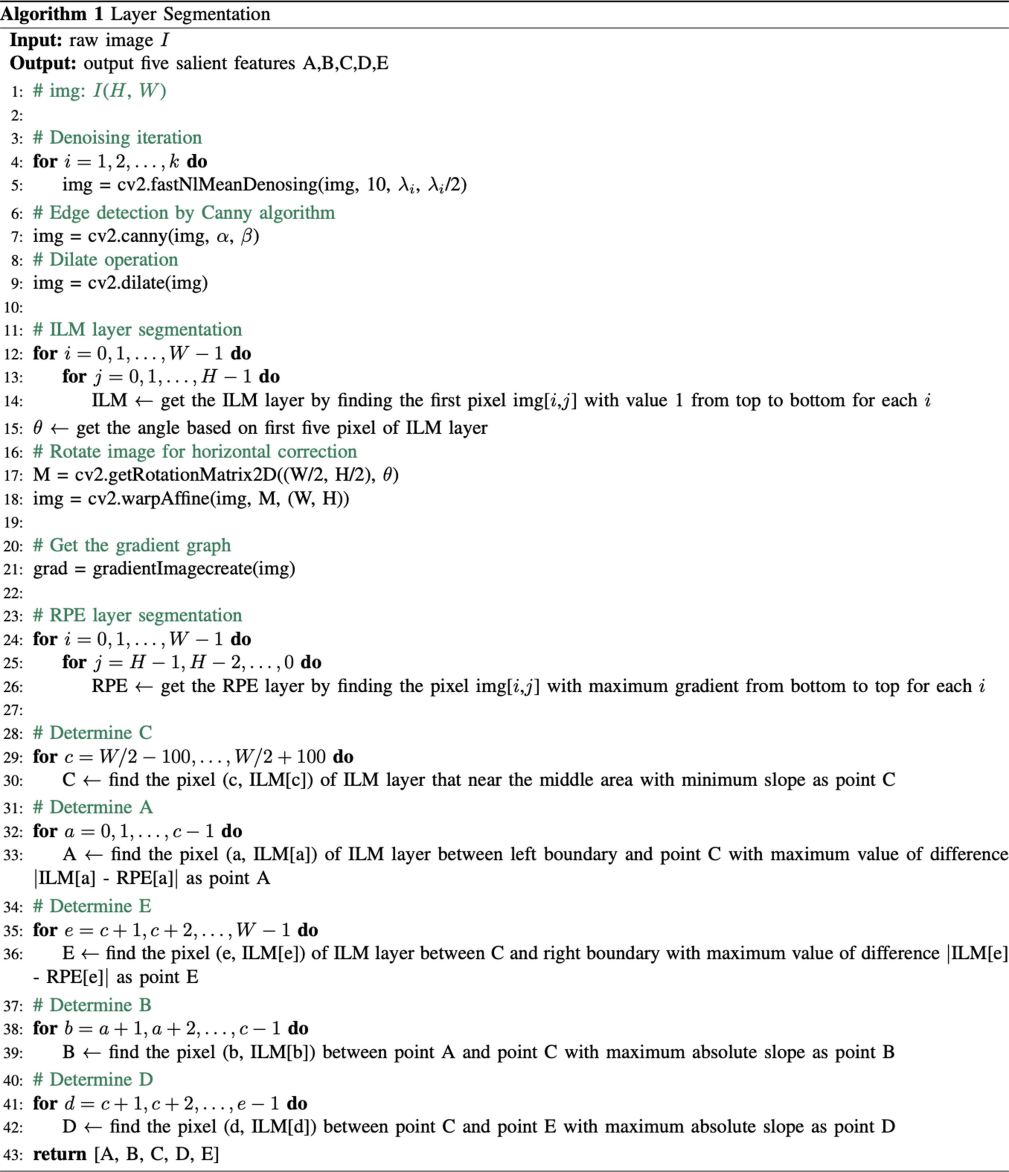


**FIGURE 1:**
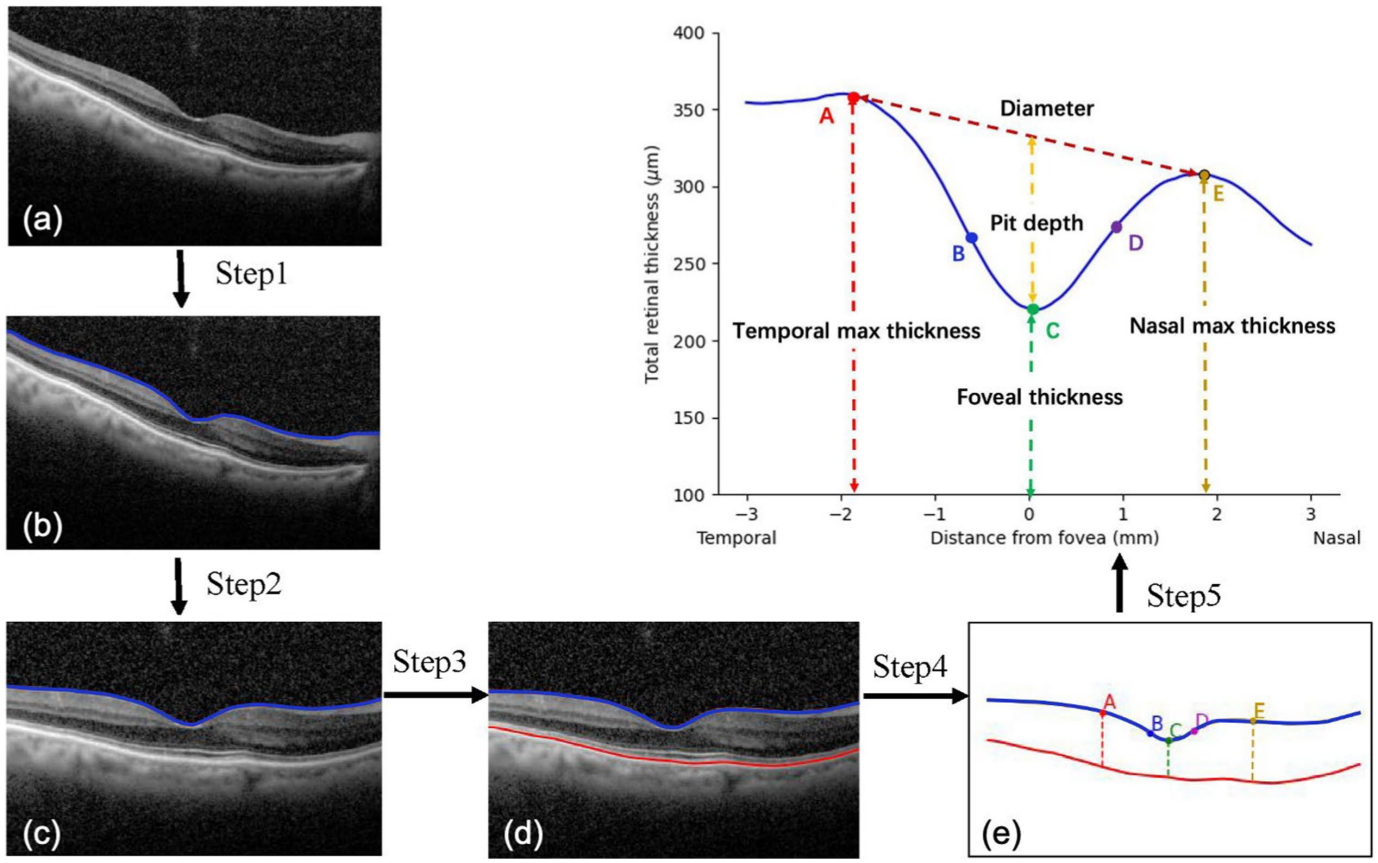
Processing of macular images. **(A,E)** A zero slope and the peak of the temporal and nasal foveal rims; **(C)** A zero slope and the center of the fovea; **(B,D)** The maximum slopes of the foveal wall.

To fully obtain the information from OCT images, morphological features of the macula were extracted and analyzed. The process of image analysis is presented in Figure 1. Firstly, irrelevant signal-to-noise was reduced by the denoising algorithm. The pixels were smoothly connected using the dilate algorithm, and the edges were detected to obtain the layered boundaries. The internal limiting membrane (ILM) layer was probed from top to bottom (Step 1). A horizontal correction was then performed based on the curve of the ILM layer to obtain a profile of the total retinal thickness (Step 2). Similarly, by creating a gradient graph to filter out the hazy features next to the retinal pigment epithelium (RPE) and highlight the RPE layer, the boundary of the retinal pigment epithelial cell layer was probed (Step 3). Five marks of the fovea were automatically recognized (Step 4). There was no slope in the temporal and nasal rims of the fovea in the horizontal meridian (Figures 1A,E). The pseudocode of this pre-processing process is in Algorithm 1. Additionally, the pit of the fovea (Figure 1C) had no slope. A maximum slope for the temporal and nasal foveal walls of the horizontal meridian was also detected (Figures 1B,D). Five salient features of the foveal pit were extracted from these five marks, including foveal thickness, pit depth, diameter, maximum thickness, and foveal slope (16, 17).

### 2.3. DL models

The models consisted of three submodules based on a DL algorithm. The first was the CNN module used to extract features from OCT images. The Second was the encoding module used to encode the feature and output the embeddings of different models. The third module was the transformer module used to fuse each model’s embeddings and predict postoperative BCVA. Specifically, Resnet-18 was selected as a CNN module whose depth was fit for this task, avoiding overfitting and poor efficiency. It flattened the output feature into a 1D vector with 512 dim. In the encoding module, the vector of the image feature became a token with 128 dim, while the vector of preoperative VA (1 dim) and external morphological feature (7 dim) became tokens with 32 dim. Since the Transformer has powerful capacities in multi-modal fusion (18, 19), we applied Transformer with multi-head self-attention to learn the dependence between different models. In the Transformer module, the token of each modal and a prediction token, which had the same size, were concatenated to a token sequence, and the prediction token represented the fusion result. Follow the vanilla Transformer architecture (20), each Transformer encoder layer contained Multi-Headed Self-Attention (MSA), Layer Normalization (LN), and Feed-Forward Net-work (FFN) blocks using residual connections. Specifically, the MSA was defined as follows:


$$MSA\left(Q,K,V\right)=\text{softmax}\left(\frac{QK^{T}}{\sqrt{d_{k}}}\right)V$$

where the Q, K and V denoted the linear result on input feature X. Based on the training dataset, the SGD optimizer was used to optimize the model to minimize the root mean square error (RMSE) loss function. We assume that the VA prediction is ${\tilde{y}}_{i}$ and true postoperative VA is $y_{i}$. The RMSE loss function is formulated as follows:


$$RMSE=\sqrt{\frac{1}{N}\sum_{i=1}^{N}{\left({\tilde{y}}_{i}-y_{i}\right)}^{2}}$$

**Figure 2:**
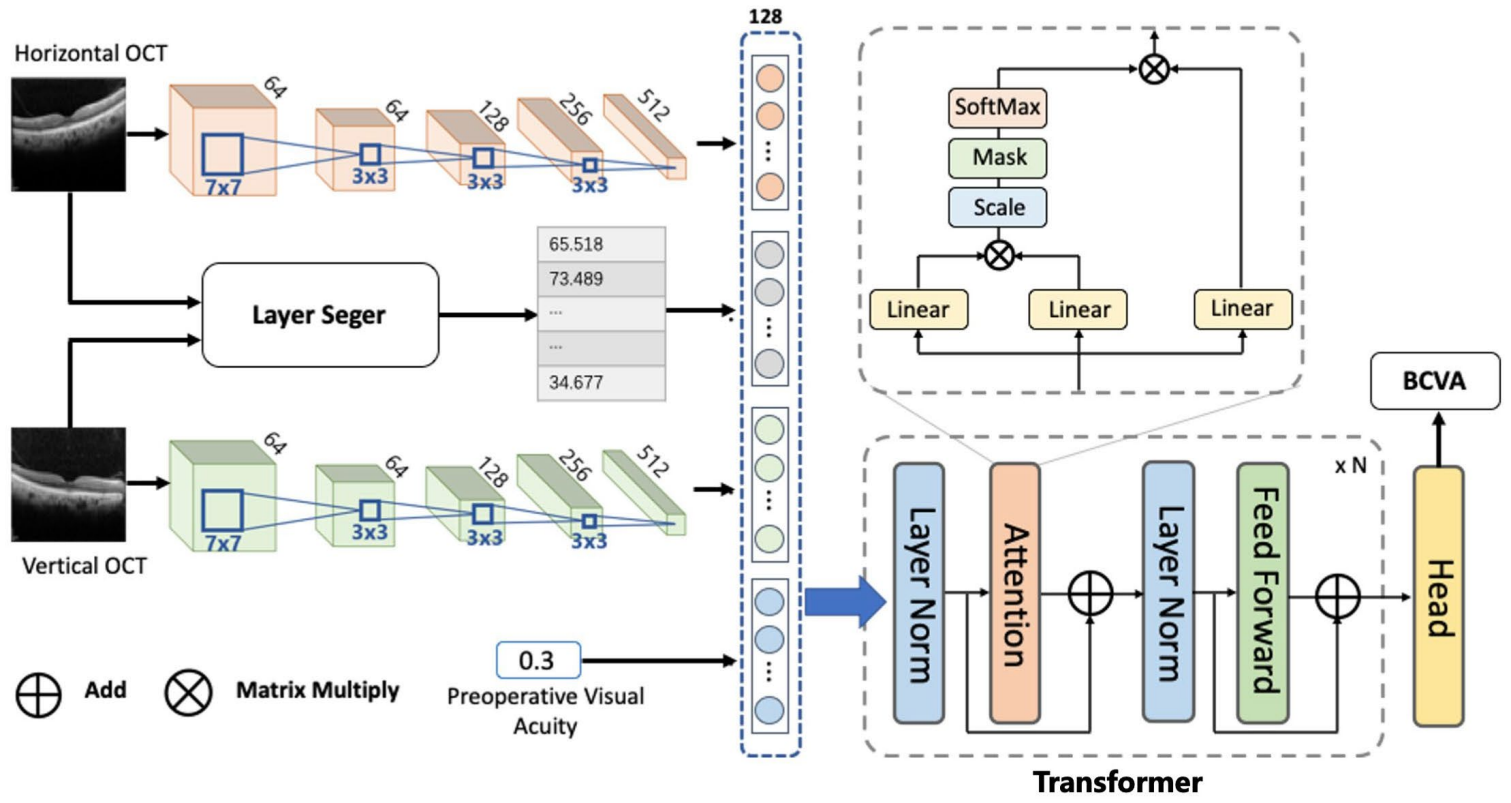
The workflow of the DL model.

The maximum number of training epochs was set to be 100. The initial learning rate was set to 0.01 and attenuated by 0.1 every 40 epochs. Figure 2 shows the workflow of the DL models.

The DL models were built based on different input information as follows:

Model I: an OCT image of horizontal B-scan,
Model II: an OCT image of vertical B-scan,
Model III: two OCT images of horizontal and vertical B-scans,
Model IV: two OCT images of horizontal and vertical B-scans, preoperative BCVA,
Model V: two OCT images of horizontal and vertical B-scans, preoperative BCVA, and the indices of macula morphological features.

### 2.4. Model performance

Two evaluation metrics, mean absolute error (MAE) and RMSE, were applied to quantitatively measure the difference between the predicted VA and the true postoperative VA. The MAE represented the mean absolute error of the prediction values, which showed the difference between the predicted and actual values. The formula for MAE was as follows:


$$MAE=\frac{1}{N}\sum_{i=1}^{N}\left|{\tilde{y}}_{i}-y_{i}\right|$$

The RMSE was the square root of the mean square error (MSE). The MSE was the mean of the squared error of the prediction values. In terms of unit agreement with the original variables, the RMSE was more interpretable.

Three general classification metrics, including precision, sensitivity, and accuracy, were used to estimate the models’ performance in predicting whether postoperative BCVA was improved by at least two lines in the visual chart (0.2LogMAR). Their methods of calculation were as follows:

Precision = TP/(TP + FP).
Sensitivity = TP/(TP + FN).
Accuracy = (TP + TN) / (TP + FP + TN + FN).
F1 = 2·Sensitivity·Precision/ (Sensitivity + Precision).

Here, TP is the true positive, FP is the false positive, TN is the true negative, and FN is the false negative. Receiver operating characteristic (ROC) curves for five models were calculated to obtain the area under the ROC curves (AUCs).

### 2.5. Statistics

Statistical analysis was performed using a commercial statistical software package (SPSS Statistics 26.0; IBM, Armonk, NY). Continuous variables were described as the mean ± standard deviation. Normal distributions for all datasets were assessed using Shapiro–Wilk normality tests. Normally distributed data were analyzed using one-way analysis of variance (ANOVA). Nonparametric data were analyzed by the Kruskal-Wallis test. The Chi-square test was used to test for categorical variables. *p* < 0.05 was considered statistically significant. The TRIPOD statement was followed.

## 3. Results

### 3.1. Patient characteristics

A total of 2,051 eyes from 2,051 patients with cataracts were included in this study. Table 1 summarizes the demographic information. No difference was found in all the clinical characteristics among the training, validation, and test datasets (*p* > 0.05).

**Table 1:** Demographic and clinical characteristics of the patients.

	Training (*n* = 1,231)	Validation (*n* = 410)	Test (*n* = 410)
Number of eyes	1,231	410	410
Female gender (%)	768 (62.4%)	242 (59.0%)	246 (60.0%)
Age (years)	69.94 ± 11.1	69.33 ± 10.78	69.35 ± 10.65
Preoperative BCVA (LogMAR)	0.66 ± 0.52	0.65 ± 0.53	0.62 ± 0.51
Postoperative BCVA (LogMAR)	0.17 ± 0.32	0.17 ± 0.32	0.17 ± 0.32
Difference between postoperative BCVA and preoperative BCVA (LogMAR)	−0.48 ± 0.45	−0.47 ± 0.43	−0.44 ± 0.4

Data were shown as the mean ± standard deviation. Abbreviation: LogMAR-logarithm of the minimum angle of resolution; BCVA-best corrected distance visual acuity.

### 3.2. Indices of macular morphological features

**Figure 3:**
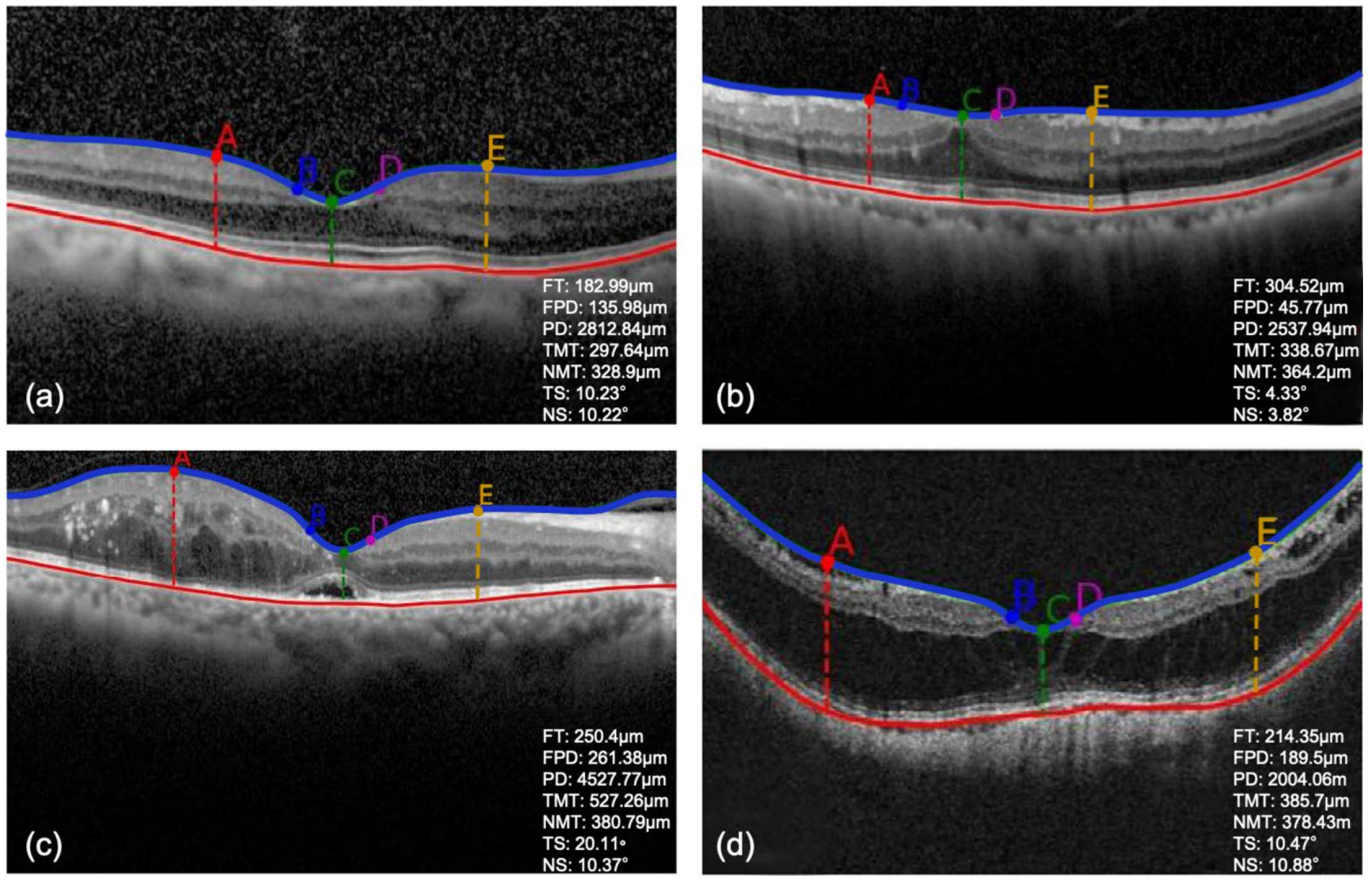
**(A–D)** The detection results of OCT images with different macular morphological changes. FT, foveal thickness; FPD, foveal pit depth; PD, pit diameter; TMT, temporal max thickness; NMT, nasal max thickness; TS, temporal foveal slope; NS, nasal foveal slope.

**Figure 4:**
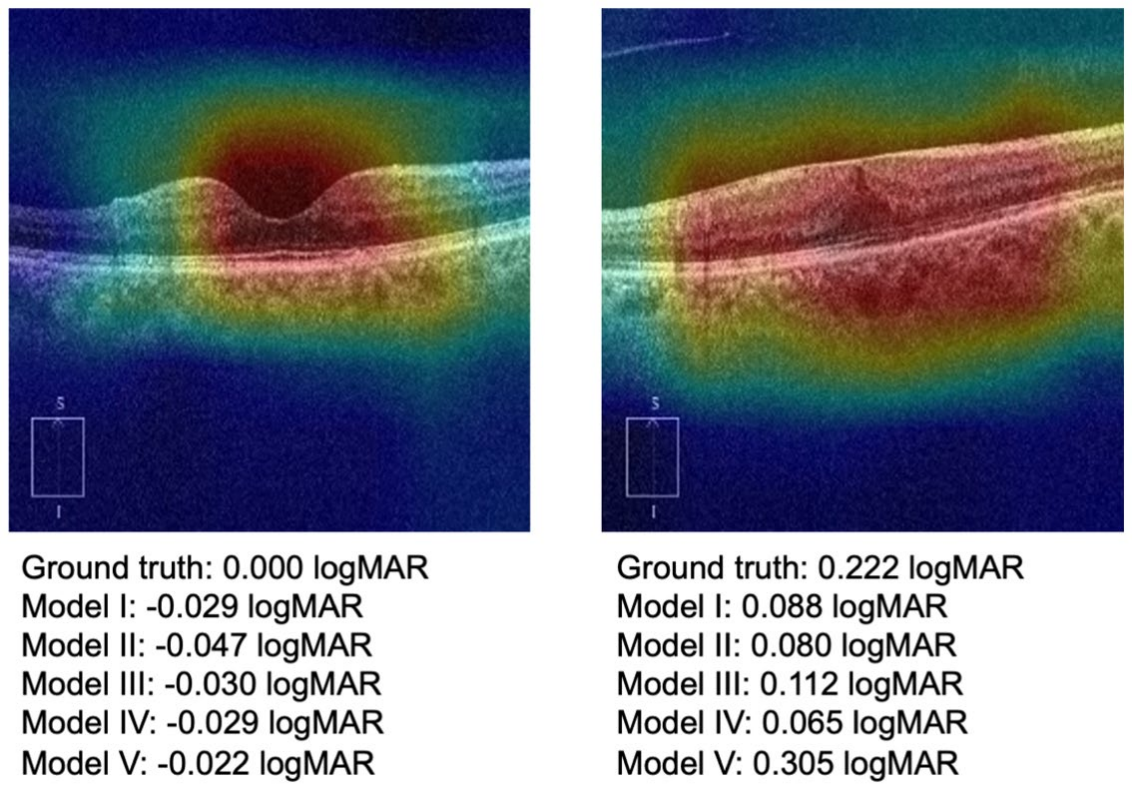
Grad-CAM results of normal macular and macular with epiretinal membrane.

Table 2 illustrates the indices of macular morphological features. There was no significant difference among the training, validation and test datasets (*p* > 0.05). Figure 3 showed the OCT images with different changes in macular morphology. It demonstrated the normal (Figure 3A), macular epiretinal membrane (Figure 3B), edema (Figure 3C), and retinoschisis (Figure 3D), and the values of indices were also shown. The Grad-CAM results were shown in Figure 4, showing the highly discriminative region of OCT scans when predicting the VA.

**Table 2:** The macular morphology detection values.

	Training	Validation	Test
Foveal thickness (μm)	277.4 ± 310.32	259.62 ± 267.34	265.16 ± 288.4
Foveal pit depth (μm)	89.53 ± 76.38	89.95 ± 74.87	89.3 ± 81.36
Foveal pit diameter (μm)	2,199.19 ± 999.12	2,204.41 ± 1004.19	2,218.88 ± 945.39
Temporal max thickness (μm)	371.64 ± 317.16	352.68 ± 276.84	358.78 ± 294.41
Nasal max thickness (μm)	370.27 ± 314.69	351.71 ± 277.75	357.4 ± 296.17
Temporal foveal slope (°)	10.06 ± 5.83	10.02 ± 6.68	10.14 ± 6.67
Nasal foveal slope (°)	10.41 ± 5.79	10.41 ± 6.97	10.47 ± 6.71

Data were shown as the mean ± standard deviation.

### 3.3. Prediction of postoperative BCVA

Table 3 presents the performance of all five models in predicting exact postoperative BCVA in the validation and test datasets. Compared with the model I-III, model IV showed better predictive performance in the validation (MAE = 0.1355logMAR, RMSE = 0.2307logMAR) and test (MAE = 0.1303logMAR, RMSE = 0.2566logMAR) datasets. When the detection and analysis of macular morphology indices were added to OCT images, the performance of model V was greatly promoted, with the lowest MAE (0.1250 and 0.1194logMAR) and RMSE (0.2284 and 0.2362logMAR) in the validation and test datasets, respectively.

**Table 3:** The performance of five models in predicting postoperative BCVA in the validation and test datasets.

Models	Validation dataset		Test dataset	
	MAE	RMSE	MAE	RMSE
I	0.1499	0.2761	0.1390	0.2624
II	0.1335	0.2504	0.1377	0.2778
III	0.1392	0.2613	0.1356	0.2730
IV	0.1355	0.2307	0.1303	0.2566
V	0.1250	0.2284	0.1194	0.2362

### 3.4. Prediction of postoperative BCVA improvement (0.2logMAR)

Table 4 presents the performance of all five models in predicting postoperative BCVA improvement (0.2logMAR) in the validation and test datasets. Compared with the model I-III, model IV showed better prediction performance in the validation (precision = 89.4%, sensitivity = 91.7%, accuracy = 85.7%, F1 = 90.5%, AUC = 0.816) and test (precision = 90.9%, sensitivity = 91.2%, accuracy = 86.6%, F1 = 91%, AUC = 0.804) datasets. Model V provided the highest precision (90.7 and 91.7%), sensitivity (93.4 and 93.8%), accuracy (88 and 89%), F1 (92 and 92.7%) and AUCs (0.856 and 0.854) in the validation and test datasets, when macular morphology indices on OCT images were detected and analyzed (*p* < 0.05).

**Table 4:** The performance of five models in predicting postoperative BCVA improvement in the validation and test dataset.

	Model	Precision (%)	Sensitivity (%)	Accuracy (%)	F1 (%)	AUC	*p* value
Validation dataset	I	90.1	86.7	83.2	88.4	0.813	0.016*
	II	90.3	89.4	85.1	89.8	0.815	0.025*
	III	88.6	92.1	85.4	90.3	0.826	0.083
	IV	89.4	91.7	85.7	90.5	0.816	0.015*
	V	90.7	93.4	88	92	0.856	–
Test dataset	I	90.8	83.4	81.2	86.9	0.802	0.001*
	II	90.5	84	81.5	87.1	0.786	<0.001*
	III	90	90.6	85.4	90.3	0.81	0.017*
	IV	90.9	91.2	86.6	91	0.804	0.003*
	V	91.7	93.8	89	92.7	0.854	–

*Statistically significant versus Model V.

## 4. Discussion

In the present study, we constructed AI prediction models for postoperative BCVA of patients with age-related cataracts based on macular OCT images and preoperative BCVA using a DL method. AI models could help doctors judge the visual outcomes of cataract surgery and aid patients in setting their surgical expectations to a reasonable level.

Ever since the first cataract surgery was performed, the evaluation of postoperative VA has been a major concern for both doctors and patients (21). Many ophthalmological examinations have been used to predict postoperative VA, such as potential acuity meter (PAM) (22–24), laser interferometer (LI) (25–27), critical flicker frequency (28–30), electrophysiological examination (31) and so on. PAM is a device combined with a slit lamp that projects a light source containing a visual chart. Light is projected through the opacified refractive media to the retina, thus providing a prediction of the patient’s postoperative VA. Gus et al. have compared the accuracy of PAM in predicting VA in patients with cataracts with different degrees of lens opacities (22). VA at 3 months post-operatively was considered to be accurate if it was between the upper and lower rows of the predicted VA. It was found that for mild to moderate cataracts, the accuracy of PAM ranged from 50 to 58.3%, for patients with severe opacities, the accuracy was only 27.8%, and for patients with extremely severe opacities, the accuracy was only 6.7%. LI projects two coherent beams from a He-Ne laser into the pupil to produce interference fringes, the width of which depends on the distance between the two beams and can be varied to correspond to the visual acuity chart by changing the width of the interference fringes (25). Similar to PAM, studies have found the accuracy of LI also needs to be enhanced (25–27). The photoreceptor cells of the retina produce a complex series of electrical responses upon light stimulation that can be recorded by visual electrophysiological examination. Based on the characteristics of its waveform, it can basically reflect the functional condition of the retina and the status of the optic nerve. Salvador et al. have performed visual electrophysiological examinations on mature cataract patients and found that the magnitude of each parameter in the visual electrophysiological examination was not affected by the degree of lens opacities (31). Analysis of waveform amplitude and latency prolongation time could indirectly reflect whether the postoperative visual prognosis was good to a certain extent. In addition, some studies have tried to explore the correlation between massive preoperative biological parameters and postoperative BCVA in patients with cataracts (15, 32–34). The preoperatively observed macular disease is found to be the factor most strongly associated with poor visual outcomes (15). However, the accuracy of the methods mentioned above needs to be further improved, and some require the subjective cooperation of patients, which is difficult in some cases. In the present study, we extracted the macular morphological features on OCT images and developed AI models to predict the postoperative VA in patients with cataracts. The prediction performance of the models was evaluated, and satisfactory prediction results were achieved.

With the rapid development of computational power and learning algorithms, AI is widely used in the field of ophthalmology, and it has also been used in predicting postoperative VA in patients with cataracts. Alexeeff et al. have compared the accuracy of three machine learning models for predicting BCVA following cataract surgery using data recorded in the electronic health system (35). Preoperative BCVA, age, and age-related macular degeneration are found to be the most critical variables in the final model, which are the key factors of our research. However, they just roughly distinguish patients with better or worse postoperative BCVA than 20/50. None of the three algorithms can accurately predict postoperative VA. Wei et al. have developed an OCT-based DL approach to predict postoperative BCVA in patients with high myopic (13). The ensemble model is found to show stably outstanding performance in internal and external test datasets. Xiang et al. have designed a system based on OCT images to predict the postoperative long-term BCVA of children with congenital cataracts (14). Six machine learning algorithms are applied. For 3-year predictions, the MAEs and RMSEs are 0.1482–0.2117 logMAR and 0.1916–0.2942 logMAR, and for 5-year predictions, they are 0.1198–0.1845 logMAR and 0.1692–0.2537 logMAR. Nevertheless, no anatomic or morphological macular features are incorporated into the study, and these data are less explicable. In our current study, we developed AI models based on preoperative OCT images and BCVA to predict the postoperative BCVA in patients with cataracts. The prediction performances of the models were further evaluated to clarify whether the model could accurately predict exact postoperative BCVA and whether the improvement (0.2logMAR) of postoperative BCVA could be predicted precisely. Promising results in the validation and test datasets were achieved, when the input information contained preoperative OCT images with horizontal and vertical B-scans, macular morphological feature indices, and preoperative BCVA. AI models that integrate large sample sizes of preoperative VA and macular OCT image morphological parameters are promising for postoperative VA prediction in patients with cataracts.

Further, the performance of the models was compared. When preoperative BCVA was added as input information, model IV performed better than Model I-III. It suggested that preoperative VA, affected by both cataract and fundus diseases, was a meaningful predictor of postoperative VA in patients with cataracts. Studies have shown that preoperative VA is related to postoperative VA to some extent, which is consistent with our study (15, 35). Model V containing preoperative OCT images with horizontal and vertical B-scans, macular morphological feature indices, and preoperative BCVA had a better performance in predicting postoperative BCVA, with the lowest MAE and RMSE, as well as the highest precision, sensitivity, and accuracy in the validation and test datasets, respectively. Geng et al. (36) have predicted the visual outcomes in patients undergoing macular hole surgery with several macular morphological parameters on OCT, including macular hole index, tractional hole index, hole form factor, area ratio factor (ARF), and volume ratio factor. ARF is found to efficiently express three-dimensional characteristics of the macular hole and has achieved good prediction results (sensitivity = 0.769, specificity = 0.786, AUC = 0.806). Sacconi et al. (37) have identified that structural OCT features are associated with BCVA outcomes in patients with type 3 macular neovascularization secondary to age-related macular degeneration after 3-year treatment with anti-VEGF injections. The presence of subretinal fluid at baseline is found to be the most significant independent negative predictor of functional outcomes. These studies have proved that the morphological abnormalities of the macula are closely associated with vision in ophthalmic diseases. In our present study, compared with a single macular OCT image, the more specific macular morphological indices, the higher accuracy of the model was revealed in predicting VA. After integrating macular morphological parameters, the prediction performance of BCVA was significantly improved, which was in consistent with the previous studies. These results suggested that OCT images, macular morphological features, and preoperative BCVA were all helpful for predicting postoperative BCVA in patients with cataracts.

Additionally, deep learning has yielded fresh perspectives on the formerly elusive correlation between retinal morphology and physiological parameters. Avinash et al. have trained a DL model to predict the refractive error from fundus images using two different datasets with high accuracy (38). For all types of refractive errors, both individual and mean attention maps, emphasizing the features that are indicative of refractive error, exhibited a distinct focus on the fovea. Yoo et al. (39) have evaluated a DL model for estimating uncorrected refractive error using retinal OCT images containing the retina and optic disc. It has been discovered that morphological features in OCT images contribute to detecting eyes with refractive errors. These studies suggest that the retina contains a wealth of previously unknown information, and that the combination of AI and retinal images may potentially lead to unexpected breakthroughs in the future.

Our study has several limitations. A study including data from multiple medical centers with a larger sample size will be helpful for AI model training. Besides, in the current study, we extracted and analyzed the external morphological features of the macula, and stratification within the retina may further improve the prediction performance of AI models. The model primarily focused on the external morphology of the macula and may not be optimal for diagnosing specific macular pathologies such as subretinal fluid or macular edema, without large-scale manually-labeled lesion data. Since the opacification of refractive media can interfere with the quality of macular OCT image and further affect the observation and extraction analysis of macular area morphology, patients with relatively good quality macular OCT images were mainly selected for this study, and the prediction accuracy of the model needs further study for patients with severe dense cataract. Further investigation and exploration are needed to integrate with more ophthalmic examinations, such as fundus photography and visual sensitivity, to establish a more informative database for a comprehensive assessment of the patient’s eyes, leading to a more accurate prediction of postoperative VA.

## 5. Conclusion

In summary, our study constructed a novel DL model to predict postoperative BCVA, showing a satisfying result in predicting postoperative BCVA in patients with cataracts. A combination AI model of OCT images, macular morphological feature indices, and preoperative BCVA was helpful for predicting postoperative BCVA in patients with cataracts.

## Data availability statement

The original contributions presented in the study are included in the article/supplementary material, further inquiries can be directed to the corresponding authors.

## Ethics statement

The studies involving human participants were reviewed and approved by the Institutional Review Board of the Second Affiliated Hospital of Zhejiang University, School of Medicine. The patients/participants provided their written informed consent to participate in this study.

## Author contributions

WX, JW, and HY created the study design. JWW and JHW performed data analysis. JWW, JHW, HY, and WX performed drafting and critical revisions of the manuscript. JWW, JHW, DC, XW, ZX, XY, SS, XL, XC, JW, HY, and WX participated in data collection. All authors contributed to the article and approved the submitted version.

## Funding

This study was funded by the National Key Research and Development Program of China (2020YFE0204400).

## Conflict of interest

The authors declare that the research was conducted in the absence of any commercial or financial relationships that could be construed as a potential conflict of interest.

## Publisher’s note

All claims expressed in this article are solely those of the authors and do not necessarily represent those of their affiliated organizations, or those of the publisher, the editors and the reviewers. Any product that may be evaluated in this article, or claim that may be made by its manufacturer, is not guaranteed or endorsed by the publisher.

## Footnotes

^1^ www.clinicaltrials.gov
